# Endothelial Rac1 is essential for hematogenous metastasis to the lung

**DOI:** 10.18632/oncotarget.3766

**Published:** 2015-05-11

**Authors:** Hongyi Yao, Wei Shi, Junsong Wu, Chengyun Xu, Jirong Wang, Yanan Shao, Ximei Wu, Zhongmiao Zhang

**Affiliations:** ^1^ Department of Pharmacy, The Second Affiliated Hospital, School of Medicine, Zhejiang University, Hangzhou, China; ^2^ Department of Pharmacology, School of Medicine, Zhejiang University, Hangzhou, China; ^3^ The First Affiliated Hospital, School of Medicine, Zhejiang University, Hangzhou, China

**Keywords:** Rac1, vascular endothelial growth factor, permeability, metastasis

## Abstract

A variety of vasoactive stimuli induce endothelial permeability through Rac1, a membrane of Rho small GTPases. Here, we determine whether tumor-secreted vasoactive stimulant through Rac1 inducing permeability contributes to hematogenous metastasis. Activation of Rac1 was assayed in human umbilical vein endothelial cells (HUVEC), transendothelial passages were measured by Transwell chambers, and hematogenously metastatic mouse model was generated by intravenous injection with Lewis lung carcinoma cells (LLC). LLC secreted abundant vascular endothelial growth factor (VEGF) in the culture media and sera of mice bearing LLC xenografts or metastatic LLC, and VEGF activated Rac1 through VEGF receptors/PI3Kβ signaling cascade, resulting in hyperoxidative stress and consequent hyperpermeability in HUVEC. Moreover, in co-culture of LLC and HUVEC, significant increases in endothelial permeability and transendothelial migration of LLC were robustly attenuated by either anti-VEGF neutralizing antibody or Rac1 knockdown in HUVEC. Finally, in metastatic mouse model, deletion of one copy of Rac1 in endothelium not only significantly attenuated LLC-induced vascular permeability, but robustly reduced the metastasis of LLC to lungs. This study supports that tumor-secreted vasoactive stimuli activate Rac1 to induce permeability and consequent transendothelial migration of tumor cells, and that loss of Rac1 function in endothelium is an effective therapeutic intervention for hematogenous metastasis.

## INTRODUCTION

Lung cancers are the leading cause of cancer deaths worldwide. Despite the local damage caused by primary lung cancers, they are only responsible for 10% of all lung cancer-related deaths; the remaining 90% persons die because of metastasis [1]. Though the molecular mechanisms underlying the metastatic processes remain poorly understood in cancer biology, a large amount of experimental evidences highlight the critical importance of the increase in vascular permeability associated with the metastasis of tumor cells [2]. Permeability of the endothelium is controlled by a variety of tumor-produced signals. The importance of tumor-secreted vascular endothelial growth factor (VEGF) in the appearance of metastasis is substantiated by the facts that: (1) the malignancy is associated with the secretion of many different isoforms of VEGF [3]; (2) VEGF isoforms augment the vascular permeability and transendothelial migration of several cancer cells to microvascular endothelial cells, that is associated with an increase in metastasis [4, 5]; (3) Most importantly, humanized monoclonal antibody against VEGF, bevacizumab, potentiates the antitumor activity of chemotherapy against systemic disease and metastases in clinics [6, 7].

Two types of endothelial junctions, adherent and tight junctions, play the critical importance in regulation of vascular permeability [8, 9]. Adherent junctions consist of transmembrane cadherins (N-, P-, and VE-cadherins), which are linked to the actin cytoskeleton by α-, β-, and γ-catenins. Tight junctions consist of the transmembrane claudins and occludin and are associated with the actin cytoskeleton via zonula occludens (ZO) proteins [8, 9]. Rho family small GTPases include three well-characterized membranes, Rho, CDC42, and Rac, all of them are important regulators of endothelial barrier properties by influencing both the endothelial actin-based cytoskeleton and the integrity of interendothelial junctions [10]. Rac1 but not Rac2 and Rac3 is highly expressed in endothelium [11], and Rac1 deficient endothelial cells fail to form lamellipodia, leading to reduced motility, permeability and impaired vascular development and angiogenesis; deletion of the Rac1 in primary endothelial cells *in vitro* revealed that Rac1 plays a central role in permeability in response to VEGF [12, 13].

Despite the clear roles of Rac1 in VEGF-mediated vascular permeability, to date, whether Rac1-mediated permeability contributes to the hematogenous metastasis of cancers remains poorly understood. Hence, by using *in vitro* co-culture and in *vivo* endothelial Rac1 knockout mouse approaches, we have investigated the potential roles of Rac1 in hematogenous metastasis of lewis lung carcinoma cells (LLC) to lung.

## RESULTS

### VEGF levels in culture media of LLC and sera of tumor-bearing mice

**Figure 1:**
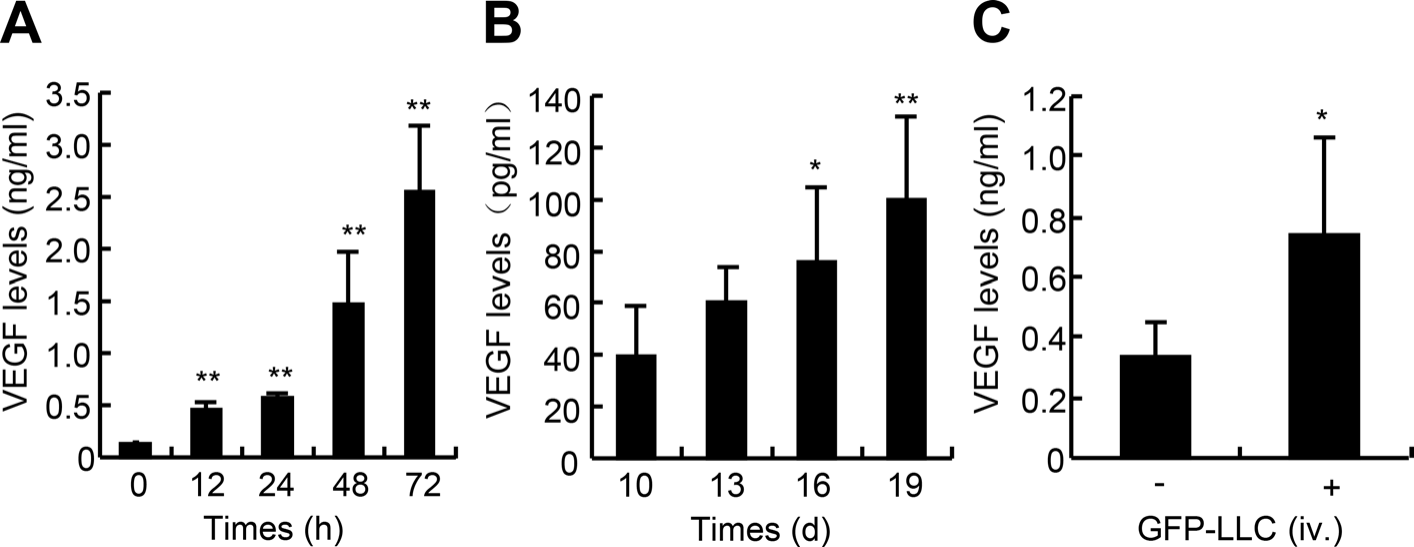
VEGF levels in the medium of LLC and sera of mice bearing either LLC xenografts or metastatic LLC **A.** VEGF levels in culture media. GFP-expressing LLC were seeded in 48-well plates at the density of 2 × 10^4^ cells/well. After pre-culture for 16-h, cells were incubated in serum-free medium for indicated times, and the supernatants were harvested for VEGF determination (*n* = 6). **B.** VEGF levels in sera of mice bearing LLC xenografts. Wild type of C57BL/6J mice were subcutaneously inoculated with the GFP-LLC (5 × 10^6^ cells/site) into both the left and right armpit of adult male C57BL/6J mice. After the tumors grew for the indicated times, the sera were harvested for VEGF determination (*n* = 7). **C.** VEGF levels in sera of mice bearing hematogenously metastatic LLC. Wild type of C57BL/6J mice were injected via caudal vein with either GFP-LLC suspension (1 × 10^6^ cells/mouse, +) or serum-free medium (−). 21-d after injection, the sera were harvested for VEGF determination (*n* = 8). *p < 0.05 versus day 10 or serum-free medium injection, **p < 0.01 versus day 0 or day 10.

To examine whether LLC produce VEGF in *in vitro* and *in vivo*, we harvested the culture media of LLC and sera from mice bearing either the LLC xenografts or hematogenously metastatic LLC. LLC expressed abundance of VEGF, and 72-h after confluence, the VEGF level in culture media reached 2.5 ng/ml that was approximately 20-fold higher than that at confluence (Figure 1A). In the mice bearing LLC xenografts, the serum VEGF level was time-dependently increased, and 19-d after inoculation it reached approximately 0.1 ng/ml (Figure 1B). In the mice bearing hematogenously metastatic LLC, 21-d after intravenous injection with LLC, the serum VEGF level reached 0.75 ng/ml that was approximately 2.0-fold higher than that in control mice (Figure 1C). Thus, LLC produce abundant VEGF both in *in vitro* and *in vivo*.

### Involvement of Rac1 in VEGF-A-induced endothelial permeability

**Figure 2:**
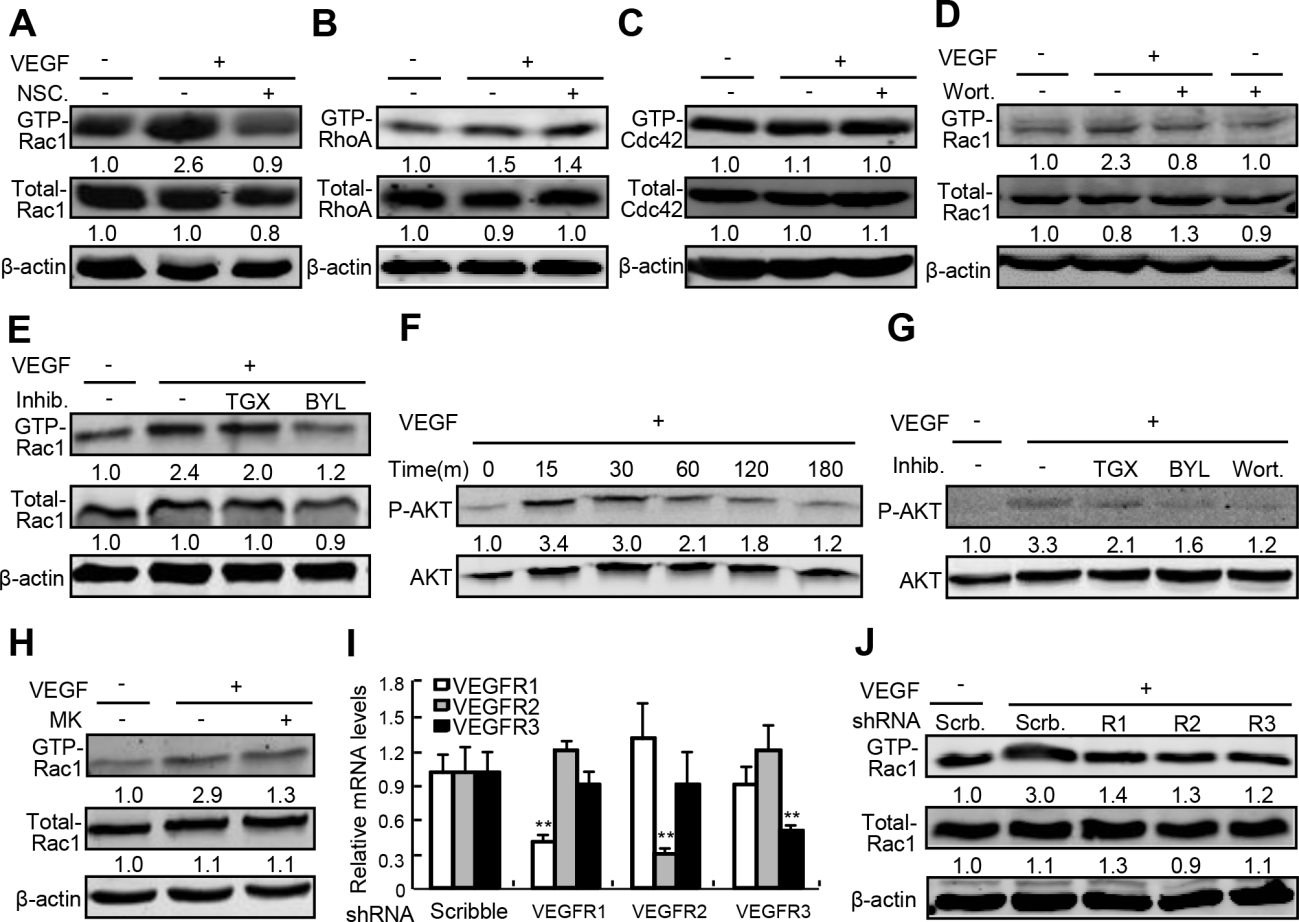
The underlying mechanism governing VEGF-A-induced Rac1 activation **A–E, H.** Activity of Rho small GTPase in response to VEGF-A in the presence or absence of NSC23766 (NSC.), wortmannin (Wort.), TGX221 (TGX), BYL719 (BYL), and MK-2206 (MK). After confluence, HUVECs were starved for 10-h, and then pretreated with NSC. at 50 μM, Wort. at 20 nM, TGX at 2 μM, BYL at 20 μM or MK at 20 μM for 2-h. Cells were further treated with recombinant VEGF-A at 50 ng/ml for 30 min and harvested for activation assays for Rho small GTPases including Rac1, CDC42 and RhoA. **F.** and **G.** Activation of AKT by VEGF and the effects of PI3K inhibitors. After confluence, HUVECs were starved for 10-h, and then pretreated with or without Wort. at 20 nM, TGX at 2 μM, BYL at 20 μM for 2-h. Then, cells were further treated with recombinant VEGF-A at 50 ng/ml for 30 min and harvested for AKT activation assays. **I.** and **J.** Involvement of VEGFRs in VEGF-A-induced Rac1 activation. After confluence, HUVECs were infected with scribble (Scrb.)-, VEGFR1-, VEGFR2-, or VEGFR3-shRNA-expressing lentiviruses, 10-h later, cells were starved for 6-h and further treated with VEGF-A at 50 ng/ml for 30 min. After that, cells were harvested for quantitative RT-PCR assays of VEGFRs mRNA and Rac1 activation assays, respectively.

To investigate the potential mechanism underlying VEGF-induced activation of Rho small GTPases and the specificity of NSC23766, a Rac1 inhibitor, in inhibition of Rac1 activity, we performed Rho small GTPases and protein kinase B (AKT or PKB) activation assays in primary human umbilical vein endothelial cells (HUVEC) treated with or without NSC23766 in the presence or absence of VEGF-A, an isoform of VEGF. VEGF-A at 50 ng/ml activated Rac1 by 1.6-fold, and NSC23766 at 50 μM not only reduced the basal levels of active Rac1 (GTP-Rac1), but also completely abolished VEGF-A-induced Rac1 activation (Figure 2A). VEGF-A activated RhoA (GTP-RhoA) by 0.5-fold and had no obvious effect on activation of CDC42 (GTP-CDC42), whereas NSC23766 at 50 μM affected neither the VEGF-A-induced activation of RhoA, nor the basal levels of CDC42 activity (Figure 2B and 2C). Phosphatidylinositol 3 kinase (PI3K) is a well-recognized upstream regulator of Rac1, to examine the roles of phosphatidylinositol 3 kinase (PI3K) and its predominant isoforms in VEGF-A-induced Rac1 activation [14–16], we treated HUVEC with wortmannin, a PI3K inhibitor with little selectivity within the PI3K isoforms, BYL719, a PI3Kα inhibitor, and TGX221, a PI3Kβ inhibitor, respectively. VEGF-A at 50 ng/ml induced Rac1 activity by 1.3-fold, and wortmannin at 20 nM almost completely negated VEGF-A-induced Rac1 activation (Figure 2D). Though TGX221 at 2 μM had no obvious effect on both basal and VEGF-A-induced Rac1 activity, BYL719 at 20 μM, almost completely attenuated VEGF-A-induced Rac1 activation (Figure 2E). To investigate the potential involvement of AKT in VEGF-induced Rac1 activation, we performed the AKT and Rac1 activation assays by using PI3K and AKT inhibitors, respectively. The phophorylation of AKT at Ser473 was time-dependently induced in response to VEGF-A at 50 ng/ml, and all the PI3K inhibitors including BYL719, TGX221, and wortamannin robustly attenuated VEGF-A-induced AKT phosphorylation (Figure 2F and 2G), however, inhibition of AKT activity by its inhibitor, MK-2206, at 20 μM had no obvious effect on VEGF-A-induced Rac1 activation (Figure 2H). Finally, to investigate the potential involvement of VEGF receptors (VEGFR) in VEGF-A-induced Rac1 activation, we generated VEGFR1-, VEGFR2- and VEGFR3-shRNA-expressing lentiviruses to knock down the expression of VEGFR1, VEGFR2, and VEGRR3, respectively. VEGFR1-, VEGFR2- and VEGFR3-shRNA-expressing lentiviruses specifically reduced their mRNA levels by 62%, 68%, and 52%, respectively (Figure 2I), whereas VEGFR1-, VEGFR2- and VEGFR3-shRNA-expressing lentiviruses negated VEGF-A-induced Rac1 activities by 54%, 57%, and 60%, respectively (Figure 2J). Thus, VEGF-A activates the Rac1 through VEGFRs/PI3Kβ signaling cascade.

### Involvement of oxidative stress in Rac1-mediated permeability in HUVEC

**Figure 3:**
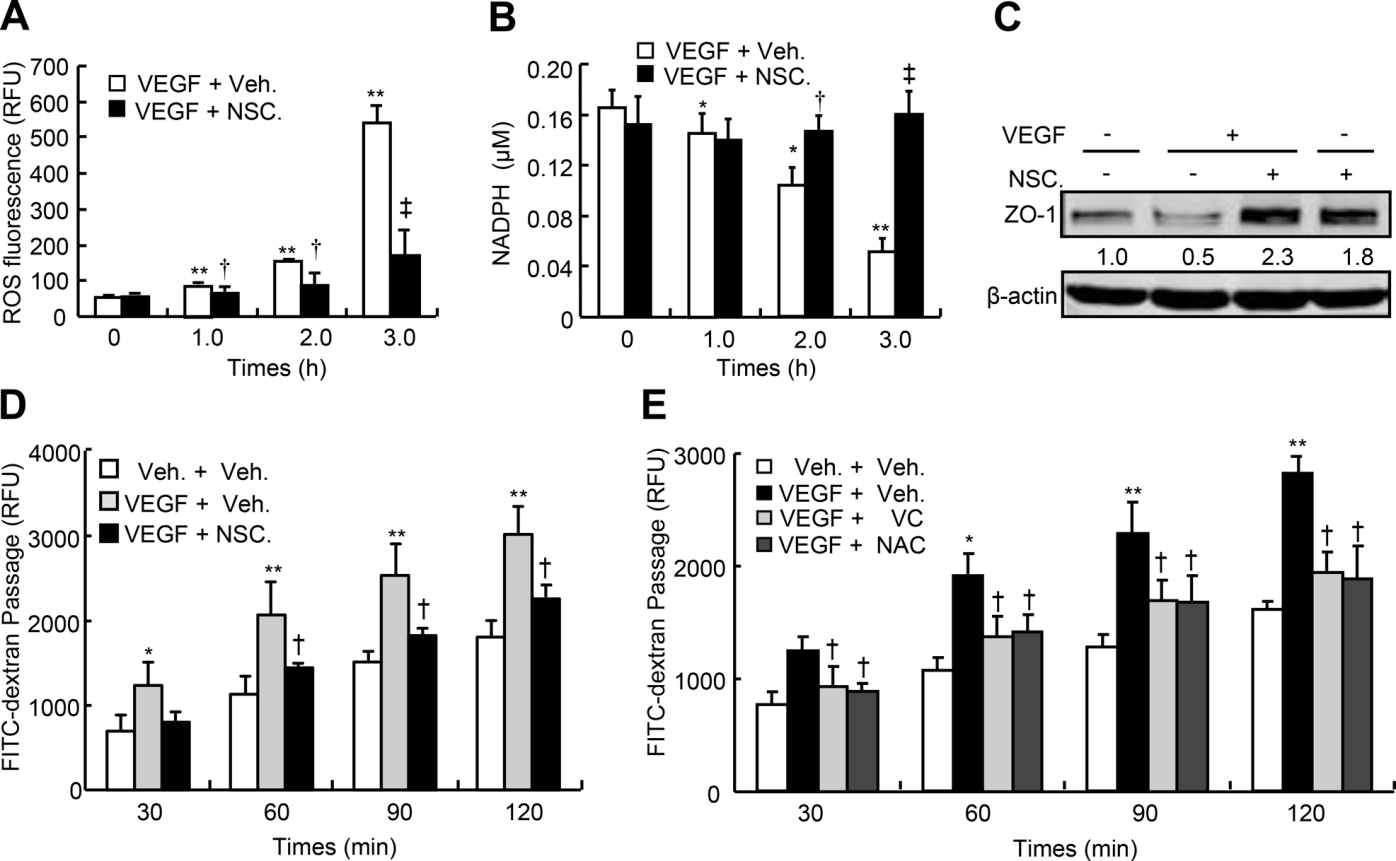
Oxidative levels, ZO-1 expression and transendothelial permeability of HUVEC in response to VEGF-A **A.** and **B.** Oxidative levels in HUVEC. After confluence, HUVEC were pretreated with or without NSC23766 (NSC.) at 50 μM for 2-h, and further incubated with VEGF-A at 50 ng/ml for indicated times. After that, cells were harvested for ROS and NADPH determination. **C.** ZO-1 expression in HUVEC. After confluence, HUVEC were pretreated with or without NSC. at 50 μM for 2-h, and further incubated with VEGF-A at 50 ng/ml for another 24-h. After that, cells were harvested for ZO-1 western blot. **D.** and **E.** HUVEC were plated in the upper well of Transwell filter at the density of 1 × 10^4^ cell/well. After confluence, cells were pre-incubated with vehicle (Veh.), NSC. at 50 μM, vitamin C (VC) at 50 μM or N-acetylcysteine (NAC) at 500 μM for 2-h, and then further treated with VEGF-A (50 ng/ml) in the presence of 10-kDa FITC-dextran (1 mg/ml) for indicated times. The fluorescent contents in the lower chamber were determined by spectrophotofluorometer (*n* = 6). Total Rac1, CDC42 or RhoA was used as an internal control for active form of Rac1, CDC42 or RhoA, respectively, and β-actin was used as an internal control for total Rac1, CDC42, RhoA and ZO-1, respectively. The mean levels that arose from cells treated without VEGF and NSC. were defined as 1, and values were described under each band. *p < 0.05 versus both vehicle treatment; **p < 0.01 versus VEGF and vehicle treatment.

To investigate the downstream events in Rac1-mediated permeability, we determined the levels of reactive oxygen species (ROS) and nicotinamide adenine dinucleotide phosphate (NADPH) and the expression of zonula occluden-1 (ZO-1), and performed permeability assays in HUVEC in response to VEGF. VEGF-A at 50 ng/ml time-dependently increased ROS levels up to 9.0-fold and reduced NADPH levels up to 33%, however, treatment with NSC23766 at 50 μM robustly reversed VEGF-A-induced increases in ROS and decreases in NADPH (Figure 3A and 3B). Meanwhile, VEGF-A at 50 ng/ml negated the expression of ZO-1 by 50%, whereas NSC23766 at 50 μM not only robustly augmented the basal level of ZO-1 (0.8-fold) but also completely reversed VEGF's effect (Figure 3C). Finally, VEGF-A at 50 ng/ml time-dependently increased the transendothelial passages of FITC-dextran, whereas inhibition of Rac1 by NSC23766 at 50 μM significantly attenuated the VEGF's effects; 120-min after treatment with VEGF, the transendothelial passages of FITC-dextran were augmented by approximately 70%, whereas NSC23766 treatment attenuated them by approximately 30% (Figure 3D). Furthermore, the anti-oxidative agents including vitamin C (VC at 50 μM) and N-acetylcysteine (NAC at 500 μM) robustly and time-dependently attenuated VEGF-A-induced transendothelial passages of FITC-dextran, 120-min after treatment, VC reduced VEGF-A-induced transendothelial passages by 33%, whereas NAC reduced them by 34% (Figure 3E). Thus, VEGF signals through VEGFR/PI3Kβ/ Rac1/ROS in regulation of endothelial junctions and subsequent endothelial permeability.

### Attenuation of transendothelial migration of LLC by knockdown of Rac1 in HUVEC

**Figure 4:**
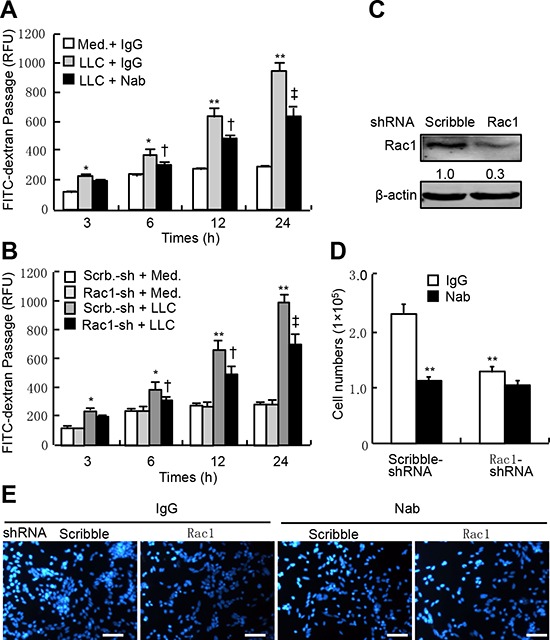
LLC-induced endothelial permeability and transendothelial migration of LLC in Rac1-knocked down HUVEC **A.** HUVEC were plated in the upper well of Transwell filter at the density of 1 × 10^4^ cell/well, after confluence, GFP-LLC (LLC) at 1 × 10^5^ cells/well or culture media (Med.) were then added, and cells were subsequently treated with anti-VEGF-A neutralizing antibody (Nab) at 500 ng/ml or control IgG in the presence of FITC-dextran (1 mg/ml) for indicated times. The fluorescent contents in the lower chamber were determined by spectrophotofluorometer (*n* = 6). **B–E.** HUVEC were infected with Rac1-shRNA (Rac1-sh) or scribble-shRNA (Scrb.-sh)-expressing lentiviruses, 48-h after infection, cells were re-plated in the upper chamber of Transwell filter at the density of 1 × 10^4^ cell/well. After tight confluence, either FITC-dextran (1 mg/ml) or GFP-LLC (1 × 10^5^ cells/well) were subsequently added in the upper chamber in the presence of Nab or IgG. After incubation for the indicated times, the fluorescent levels in the lower chamber were determined by spectrophotofluorometer, and the cells migrating to the lower chamber were counted and stained with DAPI (each *n* = 6). *p < 0.05, **p < 0.01 versus IgG treatment without LLC, infection with scribble-shRNA without LLC, or infection with scribble-shRNA without Nab; ^†^*p* < 0.05, ^‡^*p* < 0.01 versus IgG treatment with LLC, infection with scribble-shRNA with LLC, or infection with scribble-shRNA with Nab.

To determine whether tumor-secreted VEGF is sufficient to induce the permeability, we performed the co-culture of HUVEC and LLC. Similar to the cells treated with VEGF-A, LLC in co-culture system produced time-dependent increases in transendothelial passages of FITC-dextran; after 24-h, the transendothelial passages of FITC-dextran were increased by approximately 2.4-fold, and this effect was attenuated by anti-VEGF-A neutralizing antibody by approximately 35% (Figure 4A). To determine the specificity of endothelial Rac1 in LLC-induced permeability, we knocked down Rac1 expression in HUVEC. Rac1-shRNA-expressing lentiviruses led to a decrease in Rac1 protein abundance by 70% (Figure 4C). In co-culture system, tumor cells produced time-dependent increases in transendothelial passages of FITC-dextran, after 24-h, the transendothelial passages were potentiated by approximately 2.6-fold, whereas knockdown of Rac1 in HUVEC attenuated this effects by approximately 30% (Figure 4B). Likewise, both knockdown of Rac1 in HUVEC and treatment with anti-VEGF-A neutralizing antibody in co-culture system attenuated the transendothelial passages of LLC by 47% and 51%, respectively; and their combination led to a further decrease in transendothelial passages of LLC by 56% (Figure 4D and 4E). Thus, LLC-secreted VEGF is sufficient to induce the endothelial permeability and transendothelial passages of themselves, and endothelial Rac1 is required for fulfilling this effect.

### Generation of mouse model with hematogenous metastasis of LLC

**Figure 5:**
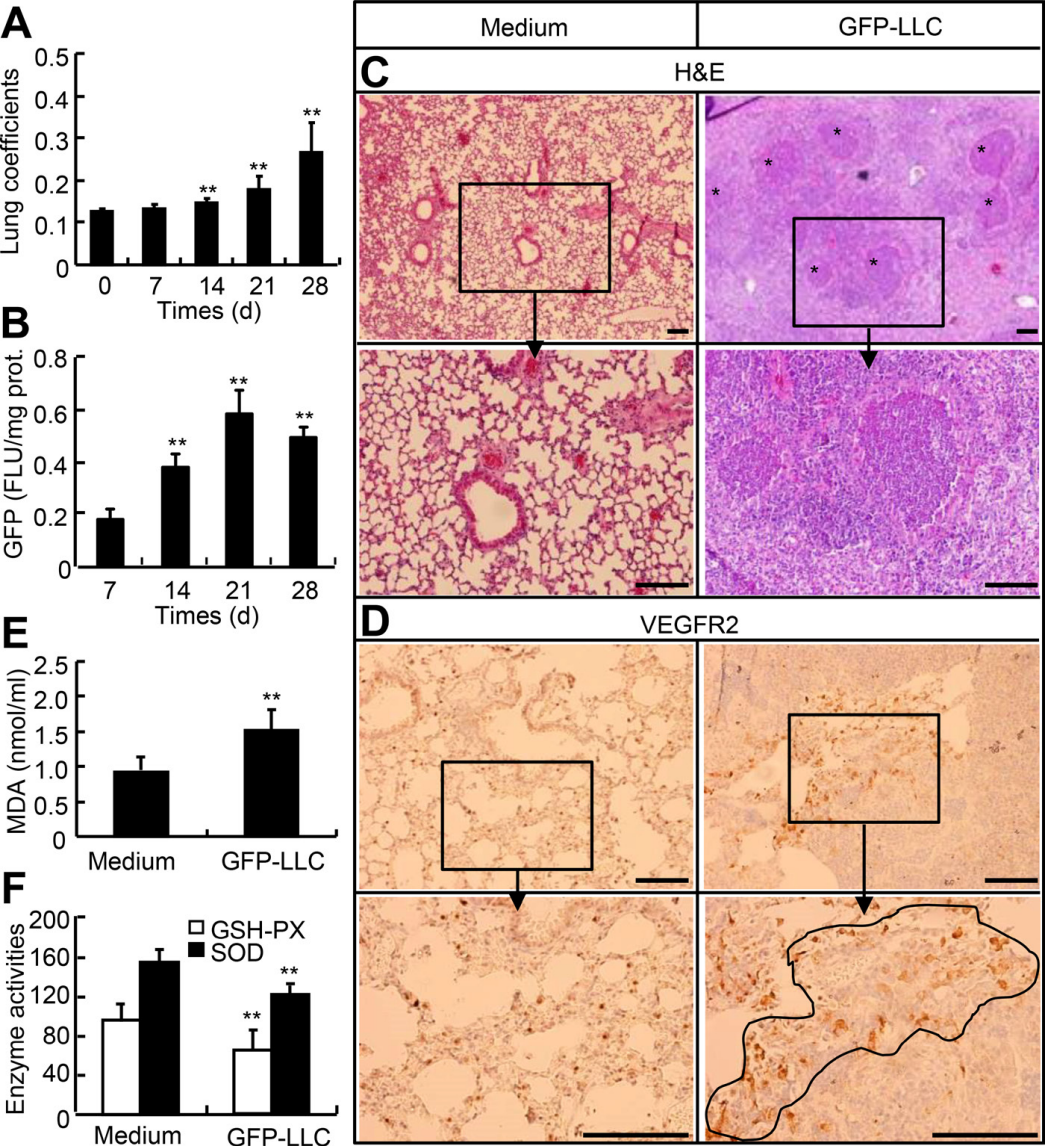
Mouse model with hematogenous metastasis of LLC to lung **A.** and **B.** Wild type of mice were injected with either GFP-LLC suspension (1 × 10^6^ cells/mouse) or serum-free medium via caudal vein. After injection, the lung and body weights were measured on day 0, 7, 14, 21, and 28, and the whole left lungs were used for homogenization and determination of GFP contents. **C–F.** On day 21, the sera were harvested for MDA, GSH-PX, and SOD determination, whereas the whole right lungs were used for histological examination and immunohistochemistry staining for VEGFR2. Each *n* = 6, *p < 0.05, **p < 0.01versus day 0, day 10, and medium, respectively.

To generate the mice bearing hematogenously metastatic LLC to lung, we intravenously injected the wild type mice with GFP-labeled LLC and monitored the growth of tumor. With the development of tumor, both the lung weight coefficients and GFP contents in lungs were significantly and time-dependently increased; 28-d after injection, the lung weight coefficients were increased by 1.1-fold; and 21-d after injection the GFP contents were increased by 2.3-fold over those 7-d after injection (Figure 5A and 5B). H&E staining and immunostaining for VEGFR2 showed that tumor tissues were different in sizes and shapes and unevenly distributed in the lungs (Figure 5C and 5D). The systemic oxidative status was evaluated by determining the serum levels of malondialdehyde (MDA), superoxide dismutase (SOD), and glutathione peroxidase (GSH-PX). The serum MDA in mice injected with GFP-LLC for 21-d was increased by 75%, whereas the serum GSH-PX and SOD were reduced by 25% and 23%, respective, as compared with in mice injected with medium alone (Figure 5E and 5F). Thus, we have succeeded in generation of hematogenously metastatic tumor model in mice, and have demonstrated that mice with hematogenously metastatic tumors are in a state of hyperoxidative stress.

### Attenuation of hematogenous metastasis by knockdown of Rac1 in endothelium

**Figure 6:**
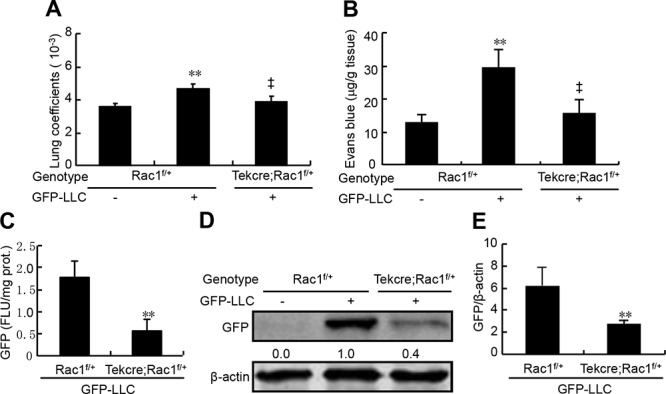
Endothelial permeability and metastasis of LLC to lungs in endothelial Rac1 knockdown mice The adult Rac1^f/+^ and TekCre;Rac1^f/+^ male mice were injected via caudal vein with either GFP-LLC suspension (1 × 10^6^ cells/mouse, +) or serum-free medium (−). 7-d after injection with GFP-LLC, mice were injected with 100 μl Evan's blue (0.5% Evans blue in sterile saline) via tail vein, 30 min after injection, the lungs were excised and the Evan's blue was extracted and determined **B.** 21-d after injection with GFP-LLC, mice were harvested for determining lung weight coefficients **A.** and GFP contents by spectrophotofluorometer **C.** and western blots **D.** and **E.**, respectively (each *n* = 8). **p < 0.01, versus Rac1^f/+^ mice injected with medium or GFP-LLC; ^‡^*p* < 0.01 versus Rac1^f/+^ mice injected with GFP-LLC.

Deletion of two copy of Rac1 in endothelium leads to embryonic lethality [13]. To determine the roles of endothelial Rac1 in hematogenous metastasis to lungs, we generated mice losing one copy of Rac1 in endothelium (TekCre;Rac1^f/+^) and their controls (Rac1^f/+^), and intravenously injected the mice with GFP-labeled LLC. 21-d after injection, GFP-LLC injection to Rac1^f/+^ mice led to an increase in the lung weight coefficients by 25% as compared with the medium injection, and GFP-LLC injection to TekCre;Rac1^f/+^ mice resulted in a decrease in lung weight coefficients by 17% over the GFP-LLC injection to Rac1^f/+^ mice (Figure 6A). 7-d after injection with GFP-LLC to mice, determination of Evan's blue showed that GFP-LLC injection led to a 1.3-fold increase in the contents of Evan's blue of lungs over control injection in Rac1^f/+^ mice, whereas deletion of one copy of Rac1 in endothelium decreased the accumulation of Evan's blue by 48% (Figure 6B). Moreover, as the metastatic tumors were inhomogeneouly distributed in the lung, we harvested the whole lungs to measure the GFP contents. Deletion of one copy of Rac1 in endothelium decreased the pulmonary GFP contents by 65% in mice injected with GFP-LLC (Figure 6C). Finally, western blot assays for GFP in whole lungs showed that deletion of one copy of Rac1 in endothelium reduced the pulmonary GFP levels by 56% in mice injected with GFP-LLC (Figure 6D and 6E). Thus, deletion of one copy of Rac1 in endothelium markedly attenuates the endothelial permeability and hematogenous metastasis of tumor cells to lungs.

## DISCUSSION

By *in vitro* cell and *in vivo* animal approaches, we have demonstrated that Rac1 possibly through VEGF/VEGFRs/PI3Kβ /Rac1/ROS signaling cascade mediates endothelial permeability and transendothelial passage of tumor cells in response to tumor-derived VEGF, and that knockdown of endothelial Rac1 attenuates hematogenous metastasis of tumor cells to lungs.

Tumor cells secrete abundant vasoactive factors that disrupt the integrity of endothelium and increase the permeability, resulting in the hematogenous metastasis of tumor cells [2]. In the present study, we identified that LLC-secreted VEGF functions as a vasoactive factor that is able to induce the permeability through Rac1. Serum murine VEGF (mVEGF) levels were increased to 0.1 ng/ml in mice bearing LLC xenografts, this value is completely consistent with the literature that has also shown the serum levels of VEGF in mice bearing LLC xenografts [17], however, in our *in vitro* experiments, we activated Rac1 in HUVEC in response to recombinant human VEGF-A (rhVEGF-A) at 50 ng/ml that is much greater than serum mVEGF levels of mice bearing either LLC xenografts or hematogenously metastatic LLC. We suggest that the bioactivity of circulating mVEGF is equivalent to that of *in vitro* rhVEGF-A in induction of endothelial Rac1 activation. The roles of Rac1 mediating VEGF signaling in endothelial permeability are well established in *in vitro* experiments [18–20], where Rac1 acts as the downstream of VEGFR2 and PI3K, and sequentially activates the signaling module composed of phospholipase Cγ (PLCγ)- AKT-endothelial nitric oxide synthase (eNOS)-extracellular regulated kinase (Erk1/2), leading to ZO-1 and occludin phosphorylation [21, 22]. Alternatively, Rac1 lies on the downstream of VEGFR2-Src-Vav2 signaling module, to either activate p21-activated protein (PAK)-mediating endothelial retraction or phosphorylate VE-cadherin. In these two pathways, all of these molecular changes lead to the endothelial opening and vascular permeability [23, 24]. Our *in vitro* data indicated that VEGF through VEGFRs, PI3Kβ activates Rac1 and subsequently induces the ROS production, and that inhibition of either Rac1 activity by its inhibitor or ROS production by antioxidants attenuates VEGF-induced endothelial permeability in primary HUVEC. Our data identify the role of not only VEGFR subtypes but also PI3K isoforms in VEGF-induced Rac1 activation and permeability. Furthermore, our data correspond to not only the above mentioned mechanism underlying VEGF-induced Rac1 activation but also the literatures showing that Rac1 lies upstream of ROS in VEGF-induced microvascular permeability [25, 26], and that VEGF signaling through VEGFRs activates Rac1 to induce endothelial NADPH oxidases-mediated ROS production in vascular permeability *in vitro* and *in vivo* [27, 28]. However, previous studies have also shown that in the absence of vasoactive stimuli, dominant negative Rac1 increases endothelial permeability and affects adherent and tight junctions [29, 30]. Moreover, Rac1-deficient endothelial cells are unable to form cellular protrusions/lamellipodia, leading to impaired cell-cell and cell-matrix interactions, and resulting in dysfunctional adhesion, motility, capillary morphogenesis as well as impaired permeability [12, 13]. These observations conflict to our results, the possible explanation is that Rac1 acts as a double-edged sword in the endothelial integrity, in the absence of vasoactive stimuli, inhibition of Rac1 activity disrupts the endothelial integrity and increases the permeability, whereas in the presence of vasoactive stimuli, attenuation of the aberrantly activated Rac1 is able to recover the endothelial integrity and permeability.

Most importantly, our data indicated that tumor cells secreted high levels of VEGF which is sufficient to induce the endothelial permeability and the transendothelial migration of tumor cells. The contribution of tumor secreted VEGF to endothelial permeability is confirmed by *in vitro* anti-VEGF-A neutralizing antibody treatments. However, in hematogenously metastatic animal model, whether the VEGF rather than other vasoactive factors derived from tumor cells triggers the metastasis to lung remains unknown. As previous studies have reported that tumor-secreted growth factors and pro-inflammatory mediators besides the VEGF such as angiopoietin-like protein-4 (Angptl4), CC-chemokine ligand 2 (CCL2), hepatocyte growth factor (HGF), stromal cell-derived factor-1 (SDF-1) and angiopoietin-1 are able to activate Rac1 and induce the endothelial permeability [31–35], we suppose that though a variety of vasoactive factors secreted by tumor cells are involved in Rac1-mediated endothelial permeability and metastasis of tumor cells, among them, VEGF alone is sufficient to increase the permeability and metastasis. This notion is further supported by previous findings that anti-VEGF-A neutralizing antibodies effectively attenuates the metastasis of tumor cells [36, 37], and administration of mice with VEGF-A alone is sufficient to induce the permeability in mice [38, 39]. Thus, based on our *in vitro* and *in vivo* observations, we speculate that tumor-produced VEGF activates Rac1, resulting in increases of permeability and consequent metastasis of tumor cells to lung in hematogenously metastatic animal model.

Beyond the importance of Rac1 in endothelial permeability and metastasis, Rac1 is aberrantly activated in a variety of tumors such as colon cancer, NSCLC, breast cancer, and generally plays an important role in cancer progression and metastasis [40]. Moreover, Rac1 is also considered to be required as a downstream molecule of many tumor-associated growth factors and pro-inflammatory mediators that are critical for the progression and malignancy of tumor [41]. For example, Rac1 acts as a downstream target of epithelial growth factor (EGF) receptor, it is recommended as a therapeutic target for gefitinib-resistant NSCLC [42]. Indeed, we have shown the striking suppression of hematogenous metastasis in mice losing of endothelial Rac1. Therefore, the involvement of Rac1 in cancer biology should not be a specific target for therapeutic intervention of tumor metastasis, but rather could be generally applicable to other cancer-associated behavior.

In summary, VEGF is secreted abundantly both in LLC culture medium and in sera of mice bearing either LLC xenografts or hematogenously metastatic LLC. Tumor cells produced VEGF activates endothelial Rac1, resulting the increases in endothelial permeability and transendothelial migration of themselves. Moreover, we have managed to identify the role of endothelial Rac1 in tumor metastasis by using a murine model of hematogenous metastasis to lung. Collectively, the present study opens up the new therapeutic potential of targeting the Rac1 pathway to control metastasis of tumor.

## MATERIALS AND METHODS

### Cell lines

Murine lewis lung carcinoma cells (LLC) were purchased from ATCC (Manassas, VA), and maintained in Dulbecco's Modified Eagle's Medium (DEME) containing 10% fetal bovine serum (Life Technologies, Inc., Carlsbad, CA). HEK293FT packaging cells (Life Technologies) for generating the lentiviruses were cultured as described previously [43]. Stably GFP-expressing LLC were generated by infecting the cells with GFP-expressing lentiviruses, and selected in the growth medium containing G418 (600 μg/ml; Sigma, St. Louis, MO) as described previously [44]. The highly GFP-expressing clones were expanded and stored for the following experiments. All cell lines were incubated at 37°C with 5% CO2.

### Isolation and maintenance of primary human umbilical vein endothelial cells

Four human placentas were obtained from uncomplicated normal term (38~40 W) pregnancy after elective cesarean section without labor, following a protocol approved by the Ethics Committee of School of Medicine of Zhejiang University. The primary HUVEC were isolated and purified as described previously [45]. After identified by immunofluorescent staining with the endothelial-specific marker CD31 and Factor VIII (purity more than 98%), HUVEC were maintained in endothelial cell medium (ECM) (Sciencell, San Diego, CA) supplemented with 20% FCS. For experiments, HUVEC were used between 2 and 5 passages.

### Mouse models bearing either LLC xenografts or hematogenously metastatic tumors

To establish the mice bearing the GFP-LLC xenografts, we subcutaneously inoculate the GFP-LLC (5 × 10^6^ cells/site) into both the left and right armpit of adult male C57BL/6J mice (SLAC Laboratory Animal Co., Ltd., Shanghai, China). After the tumors grew for the indicated times, the sera were harvested for VEGF determination.

Rac1^f/f^ mice were gifted from Dr. David A. Williams [46], and TekCre mice were purchased from MARC of Nanjing University (Nanjing, China). Rac1^f/f^ and TekCre mice with C57BL/6j background were mated to generate the Rac1^f/+^ and TekCre;Rac1^f/+^ mice for the following experiments. To establish the mouse model with hematogenously metastatic lung cancer, we injected the adult wild type or genetically modified male mice via caudal vein with either GFP-LLC suspension (1 × 10^6^ cells/mouse) or vehicle (serum-free medium). 7-d after injection, mice were subjected to *in vivo* permeability assay, and 21-d after injection, mice were subjected to determining serum VEGF levels and pulmonary GFP contents. Because the tumor was inhomogeneously distributed in lungs, the whole lungs were used for determination of GFP by western blots and spectrophotofluorometer, respectively. The lung weight coefficient was calculated by the lung weight dividing by body weight. All the mice were housed at Zhejiang University Animal Care Facility according to the institutional guidelines for laboratory animals and the protocol was approved by the Zhejiang University Institutional Animal Care and Use Committee.

### Generation of lentiviruses expressing GFP, Rac1- and VEGFR-shRNA

For construction of lentiviral shRNA-expressing vectors, the synthesized complementary oligonucleotides were annealed and inserted into the *HpaI* and *NotI* sites of pSicoR-GFP, and the integrity of the shRNA-expressing constructs were verified by a sequencer. 293FT cells were transfected with pSicoR-GFP-Rac1-shRNA, pSicoR-GFP-Scribble-shRNA (Scrib.) or pSicoR-GFP (for lentiviruses expressing GFP only) in combination with pMDL, pRSV, pVSV-G in the presence of lipofectamine 2000 reagents (Life Technologies) as described previously [43, 47]. 72-h after transfection, lentiviral supernatants were harvested and concentrated by ultracentrifugation, the viruses with titer more than 1 × 10^6^ cfu/ml were used for infection. The sense and antisense sequences of oligonucleotide for scribble-shRNA, Rac1-shRNA, VEGFR1-shRNA, VEGFR2-shRNA, and VEGFR3-shRNA were listed in the supplementary data (Table S1). The knockdown efficiency of Rac1 and VEGFRs were examined by western blots and quantitative RT-PCR, respectively.

### RNA isolation and quantitative RT-PCR

Total RNA was isolated from HUVEC by using a Trizol reagent (Takara Biotechnology Co., Ltd., Dalian, China) according to the manufacturer's instructions. 5 μg total RNA in a volume of 20 μl was reversely transcribed by using SuperScript III reagent (Life Technologies) and the oligo-(deoxythymidine) primers. After the termination of cDNA synthesis, each reaction mixture was diluted with 80 μl Tris-EDTA buffer. Messenger RNA levels of target genes were determined by quantitative RT-PCR as described previously [43]. The relative amounts of the mRNA levels of the target genes were normalized to the glyceraldehyde-3-phosphate dehydrogenase (GAPDH) levels, respectively, and the relative difference in mRNA levels was calculated by 2^−ΔΔCt^ method. The sense primers for quantitative RT-PCR of VEGFR1, VEGFR2, and VEGFR3 were 5′-TTCAAATGACCTGGAGTT-3′, 5′-ACG GACAGTGGTATGGTT-3′, and 5′-AGCCATTCATCAAC AA-GCCT-3′, respectively; while the antisense primers were 5′-ATTAGAGTGGCAGTGAGG-3′, 5′-CACAGACT CCCTGCTTTT-3′, and 5′-GGCAACAGCTGGATGTCA TA-3′, respectively.

### Determination of VEGF levels in culture supernatants and sera by ELISA

For determination of VEGF levels in culture media, GFP-expressing LLC were seeded in 48-well plates at the density of 2 × 10^4^ cells/well. After pre-culture for 16 h, cells were incubated in serum-free medium for indicated times, and the supernatants were harvested for VEGF determination. The VEGF levels both in culture media and sera were measured by Murine VEGF ELISA Development Kit (900-K99, Pepro Tech, Inc., Rocky Hill, NJ) according to the manufacturer's instructions.

### Rac small GTPases activation assays and western blots

After confluence, HUVEC were starved for 10-h and then pretreated with Rac1 inhibitor, NSC23766 (Tocris Bioscience, Bristol, UK), at 50 μM, PI3K inhibitor, wortmannin (Tocris Bioscience), at 20 nM, PI3Kα inhibitor, BYL719 (Selleck Chemicals, Houston, TX) at 20 μM, PI3Kβ inhibitor, TGX221 (Selleck Chemicals), at 2 μM for 2-h. Alternatively, when cells reached ~60% confluence, they were infected with lentivirally expressing shRNA for 24-h. Then, cells were further treated with inhibitors and human recombinant VEGF-A (R&D system, Minneapolis, MN) at 50 ng/ml for indicated times for AKT activation assays, 30 min for Rho small GTPase activation assays, or 24-h for ZO-1 western blots, and were harvested for preparing the cell lysates. After centrifugation, the supernatants were subjected to AKT and ZO-1 western blots or activation assays of Rho small GTPases including Rac1, CDC42 and RhoA by using Active GTPase Pull-Down and Detection Kits (Pierce Biotechnology, Rockford, IL) as described previously [48, 49]. Total and active forms (GTP binding form) of Rac1, CDC42 or RhoA were detected by western blots, Total Rac1, CDC42 or RhoA was used as an internal control for active form of Rac1, CDC42 or RhoA, respectively, and β-actin (Santa Cruz biotechnologies, Santa Cruz, CA) was used as an internal control for total Rac1, CDC42, RhoA and ZO-1 (Santa Cruz Biotechnologies), respectively. The semi-quantitative analysis was performed by using ImageJ software (NIH, http://rsb.info.nih.gov/ij/download.html). The mean levels that arose from cells treated without VEGF and NSC23766 were defined as 1, and values were described under each band.

### *In vitro* and *in vivo* oxidative stress assays

When HUVEC reached 90% confluence, cells were starved for 4-h and incubated in 37°C for 20 min with or without 2′, 7′-Dichlorodihydrofluorescein diacetate (DCFH-DA) which was diluted in ECM (1:1000), after washed with serum-free ECM, cells were treated with VEGF-A at 50 ng/ml and NSC23766 at 50 μM for indicated times and subjected to the determination of NADPH/NAD and ROS by EnzyChrom™ NADP/NADPH assay kit (BioAssay Systems, Hayward, CA) and ROS assay kit (Beyotime Biotechnology, Nantong, China) according to the manufacturer's instructions. For *in vivo* oxidative stress assays, mice bearing the hematogenously metastatic tumors for 21-d were sacrificed for serum isolation and determining the levels of serum superoxide dismutase (SOD), malondialdehyde (MDA), glutathione peroxidase (GSH-PX) by respective assay kits (Nanjing Jiancheng, Nanjing, china).

### *In vitro* and *in vivo* permeability assays

In VEGF-A-induced *in vitro* permeability assays, HUVEC were plated in the upper well of Transwell filter (0.4 μm, 12-mm diameter, Corning Inc., Corning, NY) at the density of 1 × 10^4^ cell/well. After tight confluence, cells were pre-incubated with vehicle, NSC23766 at 50 μM, vitamin C at 50 μM or N-acetylcystein at 500 μM for 2-h, and then treated with VEGF-A (VEGF165, 50 ng/ml) and NSC23766 in the presence of 10-kDa FITC-dextran (1 mg/ml, Life technologies) for indicated times. After that, the fluorescent levels in the lower chamber were determined by spectrophotofluorometer (excitation 485 nm, emission 520 nm).

In tumor cells-induced permeability assays, HUVEC were infected with Rac1-shRNA or Scribble-shRNA-expressing lentiviruses, 48-h after infection, cells were re-plated in the upper chamber of Transwell filter (0.4 μm for FITC-dextran passage, 8 μm for GFP-LLC passages) at the density of 1 × 10^4^ cell/well. After tight confluence, either FITC-dextran (1 mg/ml) or GFP-LLC (1 × 10^5^ cells/well) were subsequently added in the upper chamber in the presence or absence of anti-VEGF-A neutralizing antibody (R&D) at 500 ng/ml. After incubation for the indicated times, the fluorescent levels in the lower chamber were determined by spectrophotofluorometer, and the cells migrating to the lower chamber were counted and stained with DAPI.

For *in vivo* permeability assays, Rac1^f/+^ and TekCre;Rac1^f/+^ mice were intraperitoneally injected with pyrilamine maleate (4 mg/kg body weight in 0.9% saline; Sigma) to inhibit endogenous histamine release. 30-min later, mice were subsequently injected via tail vein with 100 μl Evan's blue (0.5% Evans blue in sterile saline; Sigma), which was allowed to circulate for further 30 min. Then, the lungs were excised and Evan's blue was extracted by immersion in formamide buffer. The amount of Evan's blue in lung was quantified by spectrometry at 620 nm.

### Histological and immunohistochemistry examination

Fresh lungs were fixed with 10% neutral formalin for 7-d and then embedded in paraffin. For determining the distribution of tumorous tissues in lungs, the paraffin sections (4 μm) were subjected to Haematoxylin & Eosin staining and immunohistochemistry staining for VEGFR2. Immunohistochemistry staining was performed by using the Histostain-Plus Kit (Kangwei Reagents, Beijing, China) as described previously [43, 50]. After sequential treatments, tissue sections were sequentially incubated with normal serum for 30 min, control IgG and primary antibodies against VEGFR2 (Cell Signaling, 1:200) at 4°C overnight, and then HRP-labeled secondary antibody for 30 min. The 3, 3-diaminobenzidine(DAB) solution was used for development of brown color to identify the expression of VEGFR2.

### Statistical analysis

Experiments were repeated at least three times. Numerical data were expressed as means ± S.D. and analyzed by one-way ANOVA and Tukey–Kramer multiple comparisons test. Differences were considered significant when *p* < 0.05. The SPSS statistical package (IBM, North Castle, NY, USA) was used.

## SUPPLEMENTARY TABLE


