# Supplementary material for: Endothelial Rac1 is essential for hematogenous metastasis to the lung
Source: Oncotarget. 2015 May 11;6(19):17501–13. doi: 10.18632/oncotarget.3766 (PMC4627324; doi:10.18632/oncotarget.3766)
Supplement: Supplementary file 1 [file oncotarget-06-17501-s001.pdf]

# SUPPLEMENTARY TABLE

**Supplementary Table S1: The sequences of oligonucleotide for lentivirally expressing shRNA**

| Gene | Sequences |
|---|---|
| ***Scribble*** | |
| **Sense:** | 5′-AACGTTCTCCGAACGTGTCACGTTTTCAAGAGAAAACGTGACACG TTCGGAGAATTTTTTC-3′ |
| **Antisense:** | 5′-AACGAGGGTCTAGCCATGGCTAA GGAGATTTCAAGAGAATCTCCTTA GCCATGGCTAGACCCTTTTTTTC -3′ |
| ***Rac1*** | |
| **Sense:** | 5′-TCGAGAAAAAATTCTCCGAACGTGTCACGTTTTCTCTTGAAAAACGTG ACACGTTCGGAGAACGTT-3 |
| **Antisense:** | 5′-TCGAGAAAAAAAGGGTCTAGCCATGGCTAAGGAGATTCTCTTGAAATCT CCTTAGCCATGGCTAGACCCTCGTT-3′ |
| ***VEGFR1*** | |
| **Target 1 sense:** | 5′-AACGCCGCCGCTTCCATTTTTCC TTTCAAGAGAAGGAAAAATGGAAGCG GCGGCTTTTTTC-3′ |
| **Target 1 antisense:** | 5′-TCGAGAAAAAAGCCGCCGCTTCCATTTTTCCTTCTCTTGAAAGGAAAAAT GGAAGCGGCGGCGTT-3′ |
| **Target 2 sense:** | 5′-AACGAAGGAC GTAACTGAAGAGGATTTCAAGAGAATCCTCTTCAGTT ACGTCCTTCTTTTTTC-3′ |
| **Target 2 antisense:** | 5′-TCGA GAAAAAAGAAGGACGTAACTGAAGAGGATTCTCTTGAAATCCTC TTCAGTTACGTCCTTCGTT-3′ |
| ***VEGFR2*** | |
| **Target 1 sense:** | 5′-AACGAAGCGGCTACCAGTCCGGATATTCAAGAGATATCCGGACTGGTAGC CGCTTCTTTTTTC-3′ |
| **Target 1 antisense:** | 5′-TCGAGAAAAAAGA AGCGGCTACCAGTCCGGATATCTCTTGAATATCCGGA CTGGTAGCCGCTTCGTT-3′ |
| **Target 2 sense:** | 5′-AACGGTCCCTCAGTGATGTAGAATTCAAGAGATTCTACATCACTGAGGGA CCTTTTTTC-3′ |
| **Target 2 antisense:** | 5′-TCGAGAAA AAAGGTCCCTCAGTGATGTAGAATCTCTTGAATTCTACATCA CTGAGGGACCGTT-3′ |
| ***VEGFR3*** | |
| **Target 1 sense:** | 5′-AACGCCAGGATGAAGACATTTGATTCAAGAGATCAAATGTCTTCATCCTG GCTTTTTTC-3′ |
| **Target 1 antisense:** | 5′-TCGAGAAAAAAGCCAGGATGAAGACATTTGATCTCTTGAATCAAATGTCT TCATCCTGGCGTT-3′ |
| **Target 2 sense:** | 5′-AACGCTCCTCATCTTCTGTAACATTCAAGAGATGTTACAGAAGATGAGGA GCTTTTTTC-3′ |
| **Target 2 antisense:** | 5′-TCGAGAAAAAAGCTCCTCATCTTCTGTAACATCTCTTGAATGTTACAGAA GATGAGGAGCGTT-3′ |
